# Correction: Exploration of the cofactor specificity of wild-type phosphite dehydrogenase and its mutant using molecular dynamics simulations

**DOI:** 10.1039/d1ra90111g

**Published:** 2021-05-12

**Authors:** Kunlu Liu, Min Wang, Yubo Zhou, Hongxiang Wang, Yudong Liu, Lu Han, Weiwei Han

**Affiliations:** Key Laboratory for Molecular Enzymology and Engineering of Ministry of Education, School of Life Science, Jilin University 2699 Qianjin Street Changchun 130012 China luhan@jlu.edu.cn; High School Attached to Northeast Normal University 506 Ziyou Road Changchun 130012 China

## Abstract

Correction for ‘Exploration of the cofactor specificity of wild-type phosphite dehydrogenase and its mutant using molecular dynamics simulations’ by Kunlu Liu *et al.*, *RSC Adv.*, 2021, **11**, 14527–14533, DOI: 10.1039/D1RA00221J.

The authors regret that an incorrect grant number was shown in the acknowledgements section of the published article. The corrected section should read:

This work was supported by the National Natural Science Foundation of China [31870201] and the Overseas Cooperation Project of Jilin Province [20200801069GH]. This work was performed at the High Performance Computing Center of Jilin University.

The Royal Society of Chemistry apologises for these errors and any consequent inconvenience to authors and readers.

## Supplementary Material

